# Small-molecule inhibition of BRD4 as a new potent approach to eliminate leukemic stem- and progenitor cells in acute myeloid leukemia (AML)

**DOI:** 10.18632/oncotarget.733

**Published:** 2012-11-27

**Authors:** Harald Herrmann, Katharina Blatt, Junwei Shi, Karoline V. Gleixner, Sabine Cerny-Reiterer, Leonhard Müllauer, Christopher R. Vakoc, Wolfgang R. Sperr, Hans-Peter Horny, James E. Bradner, Johannes Zuber, Peter Valent

**Affiliations:** ^1^ Ludwig Boltzmann Cluster Oncology, Vienna, Austria; ^2^ Department of Internal Medicine I, Division of Hematology and Hemostaseology, Medical University of Vienna, Austria; ^3^ Cold Spring Harbor Laboratory, Cold Spring Harbor, NY, USA; ^4^ Department of Pathology, Medical University of Vienna, Austria; ^5^ Department of Medical Oncology, Dana-Farber Cancer Institute, Harvard Medical School, Boston, MA, USA; ^6^ Institute of Pathology, Ludwig-Maximilians-University, Munich, Germany; ^7^ Research Institute of Molecular Pathology (IMP), Vienna, Austria

**Keywords:** AML, leukemic stem cells, BRD4, JQ1, targeted therapy

## Abstract

Acute myeloid leukemia (AML) is a life-threatening stem cell disease characterized by uncontrolled proliferation and accumulation of myeloblasts. Using an advanced RNAi screen-approach in an AML mouse model we have recently identified the epigenetic ‘reader’ BRD4 as a promising target in AML. In the current study, we asked whether inhibition of BRD4 by a small-molecule inhibitor, JQ1, leads to growth-inhibition and apoptosis in primary human AML stem- and progenitor cells. Primary cell samples were obtained from 37 patients with freshly diagnosed AML (n=23) or refractory AML (n=14). BRD4 was found to be expressed at the mRNA and protein level in unfractionated AML cells as well as in highly enriched CD34^+^/CD38^−^ and CD34^+^/CD38^+^ stem- and progenitor cells in all patients examined. In unfractionated leukemic cells, submicromolar concentrations of JQ1 induced major growth-inhibitory effects (IC_50_ 0.05-0.5 μM) in most samples, including cells derived from relapsed or refractory patients. In addition, JQ1 was found to induce apoptosis in CD34^+^/CD38^−^ and CD34^+^/CD38^+^ stem- and progenitor cells in all donors examined as evidenced by combined surface/Annexin-V staining. Moreover, we were able to show that JQ1 synergizes with ARA-C in inducing growth inhibition in AML cells. Together, the BRD4-targeting drug JQ1 exerts major anti-leukemic effects in a broad range of human AML subtypes, including relapsed and refractory patients and all relevant stem- and progenitor cell compartments, including CD34^+^/CD38^−^ and CD34^+^/CD38^+^ AML cells. These results characterize BRD4-inhibition as a promising new therapeutic approach in AML which should be further investigated in clinical trials.

## INTRODUCTION

Acute myeloid leukemia (AML) is a stem cell-derived hematopoietic malignancy characterized by uncontrolled proliferation and accumulation of myeloblasts in the bone marrow (BM), blood, and other organs. The clinical course and prognosis in AML vary, depending on age, the biology and category of the disease, cytogenetic features and number and types of deregulated genes [1-6]. In a subset of patients, cytogenetic and/or molecular features are indicative of a more favorable prognosis. When treated with repeated chemotherapy cycles or/and hematopoietic stem cell transplantation (SCT), these patients have a relatively good prognosis and many of them enter long-term disease-free survival [1-8]. However, not all patients with AML have a suitable donor or are eligible for SCT. In other patients, the response to chemotherapy is poor or short-lived. For AML patients who relapse or have resistant disease, therapeutic options are limited. Current research is seeking novel drug targets and novel, more potent, drugs for these poor-risk patients [9-13].

One key event in oncogenic transformation in AML is the corruption of myeloid cell-fate programs resulting in the generation of aberrantly self-renewing cells, the so-called leukemic stem cells (LSC), which maintain and propagate the disease and are often resistant to conventional chemotherapy [14-16]. Notably, in AML and other myeloid leukemias, the malignant clone is organized hierarchically with more mature cells programmed to undergo apoptosis after a variable number of cell divisions, and immature primitive cells that have self-renewing and leukemia-propagating capacity [14-18]. Although the exact phenotype of LSC in AML remains uncertain, several studies have suggested that in various AML subtypes, the NOD/SCID mouse-repopulating LSC reside within a CD34^+^/CD38^−^ fraction of the leukemic clone [16-21]. Other studies have shown that NSG mouse-repopulating AML stem cells reside in both the CD34^+^/CD38^+^ and the CD34^+^/CD38^−^ fraction of AML cells, or even in CD34^−^ subfractions of the clone [22,23]. Clinically, the LSC concept is of great importance and may have prognostic and therapeutic implications [24-32]. Notably, strategies aimed at terminating aberrant self-renewal or survival of LSC are considered key to the development of more effective therapies [26-32].

In an effort to systematically probe genes involved in chromatin regulation as potential therapeutic targets, we have recently developed an unbiased screen approach, combining AML mouse models and new *in vivo* RNAi technologies. Through this approach we were able to identify the epigenetic ‘reader’ Bromodomain-containing 4 Protein (BRD4) as a new potential target in AML [33]. Inhibition of BRD4 using BRD4-specific RNAi or JQ1, a BET bromodomain inhibitor that blocks BRD4-binding to acetylated histones, showed profound antileukemic effects in AML mouse models as well as in various human AML cell lines and in primary leukemic cells obtained from AML patients [33].

In the present study, we extended these analyses to various subtypes of AML as well as to AML LSC. The specific aim of our study was to evaluate BRD4-inhibition as a potential therapeutic approach to target and eliminate LSC in AML. To address this question, we analyzed the effects of JQ1 on primary neoplastic stem- and progenitor cells obtained from patients with freshly diagnosed or refractory AML. In addition, we asked whether JQ1 would synergize with conventional cytostatic drugs to produce synergistic anti-leukemic effects in AML.

## RESULTS

### BRD4 is expressed in AML cells including CD34^+^ stem^−^ and progenitor cells

**Figure 1:**
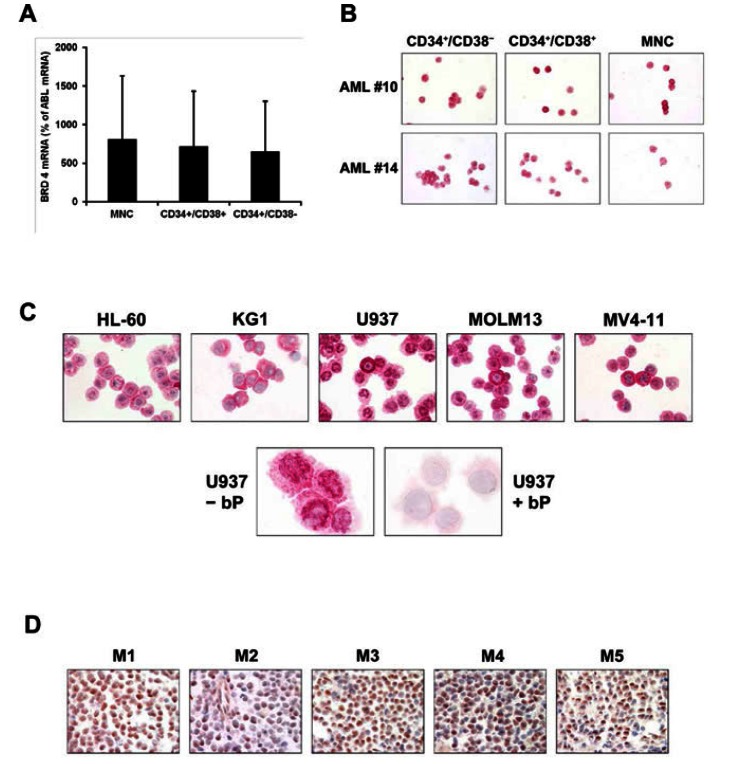
Expression of BRD4 in leukemic cells in acute myeloid leukemia (AML) A: Highly purified (sorted) CD34^+^/CD38^−^ and CD34^+^/CD38^+^ stem and progenitor cells of 9 patients with AML were subjected to RNA isolation and qPCR as described in the text. BRD4 mRNA levels are shown as percent of ABL mRNA levels. Results are expressed as mean±S.D. of 9 donors. B: Detection of the BRD4 protein in the cytoplasm and in the nuclei of CD34^+^/CD38^−^ (left panels) and CD34^+^/CD38^+^ stem- and progenitor cells (middle panels) purified from mononuclear cells (MNC) by cell sorting (purity >98%) in 2 patients with AML (numbers refer to patients shown in Table 3). Immunocytochemistry was performed on cytospin slides using an anti-BRD4 antibody. The staining reaction in the total (non-sorted) MNC fraction is also shown (right panels). C: Expression of BRD4 in AML cell lines (HL60, KG-1, U937, MOLM13, MV4-11) as determined by indirect immunocytochemistry using an anti-BRD4 antibody. In the lower panel, the anti-BRD4 antibody was preincubated with control medium (-bP; lower left panel) or with a BRD4-specific blocking peptide (+bP, lower right panel) before being applied on U937 cells. D: Immunohistochemical detection of BRD4 in AML cells in bone marrow sections obtained from patients with AML (FAB categories M1, M2, M3, M4, and M5). Again, BRD4 was detected in the cytoplasm as well as in the nuclei of AML blasts. Indirect immunohistochemistry was performed using an anti-BRD4 antibody.

As assessed by qPCR analysis, BRD4 mRNA was found to be expressed in highly enriched sorted CD34^+^/CD38^+^ AML progenitor cells and CD34^+^/CD38^−^ stem cells (Figure 1A). In addition, all AML cell lines examined (HL60, U937, KG1, MV4-11, MOLM-13) were found to express BRD4 mRNA (not shown). Expression of the BRD4 protein in AML cells was examined by ICC and IHC. As assessed by ICC, BRD4 was found to be expressed in primary AML cells (blasts) in all donors without negative subpopulations (Figure 1B). More importantly, we found that in all donors examined, the CD34^+^/CD38^+^ and the CD34^+^/CD38^−^ stem- and progenitor cells express the BRD4 antigen without negative subpopulations (Figure 1B). No differences in BRD4 expression were seen when comparing different FAB or WHO subtypes of AML. In addition, all AML cell lines tested were found to stain positive for BRD4 (Figure 1C). BRD4 was found to be expressed in both the cytoplasmic compartment and nuclear compartment of leukemic cells in all patients and all cell lines tested (Figure 1B and 1C), and the same was found when normal BM cells or cord blood cells were analyzed (not shown). Preincubation of the anti-BRD4 antibody with a specific blocking peptide resulted in a negative stain (Figure 1C). Corresponding results were obtained by IHC. Again, BRD4 was found to be expressed in the nuclear and cytoplasmic compartment of leukemic cells in all donors and all AML variants tested (Figure 1D). In the normal BM, BRD4 was also expressed in myeloid progenitor cells as well as in megakaryocytes. However, compared to the leukemic marrow, BRD4 expression appeared to be more restricted to the nuclear compartment of myeloid cells. Table 1 shows the distribution of BRD4 in the various cellular compartments in AML and in control BM sections. Together, our data show that BRD4 is expressed in both the cytoplasm and in the nuclei of AML blasts and AML LSC.

**Table 1:** Cellular distribution of BRD4 in bone marrow (BM) sections in AML and controls

	cytoplasmic BRD4 staining			nuclear BRD4 staining		
Cell type	normal/ reactive BM	primary AML	relapsed/refractory AML	normal/ reactive BM	primary AML	relapsed/refractory AML
Megakaryocytes	+/−	+/−	+	+	+	+
Myeloblasts	n.d.	+/−	+	n.d.	+/−	+
Myeloid progenitor cells	+/−	+	+	+	+	+
Neutrophil granulocytes	+/−	+/−	+/−	+/−	+/−	+/−
Eosinophil granulocytes	−	+/−	+/−	+/−	+/−	+/−
Erythroid cells	−	−	−	−	−	−
Lymphocytes	n.d.	+/−	+/−	+/−	+/−	+/−

Bone marrow sections were stained with an anti-BRD4 antibody as described in the text.

Score: −, negative; +/−, weakly positive or positive in a smaller subset of cells; +, mostly positive.

Abbreviations: BM, bone marrow; AML, acute myeloid leukemia; n.d., not determined.

### The BRD4-targeting drug JQ1 inhibits proliferation of leukemic cells in freshly diagnosed and relapsed or refractory AML

**Figure 2:**
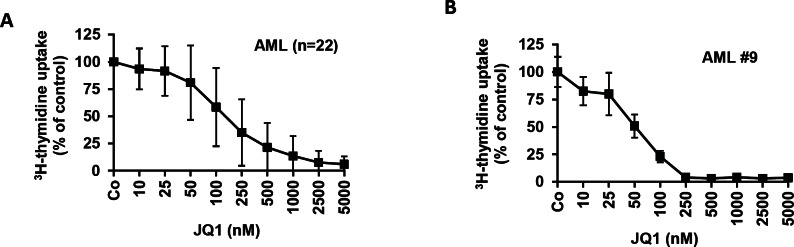
Effects of JQ1 on proliferation of AML cells A: Isolated AML blasts of 22 patients with AML were incubated in control medium (Co) or medium containing various concentrations of JQ1, as indicated, at 37°C and 5% CO_2_ for 48 hours. Then, uptake of ^3^H-thymidine was measured. Results are expressed as percent of medium control and show the mean±S.D. values from these 22 patients. B: AML blasts from patient #9 (in Table 3) were incubated in control medium or medium containing various concentrations of JQ1 (as indicated) at 37°C and 5% CO_2_ for 48 hours. Then, uptake of ^3^H-thymidine was measured. Results are expressed as percent of control and show the mean±S.D. of triplicates.

Confirming our previous data [33] the BET bromodomain inhibitor JQ1 was found to block ^3^H-thymidine uptake and thus proliferation in unfractionated primary AML blasts. In particular, JQ1 was found to inhibit the proliferation of leukemic cells in 27/28 AML patients tested, with reasonable IC_50_ values (<1 μM) (Table 2). Anti-leukemic effects of JQ1 were seen in all patients with primary, untreated AML (n=18), and in 9/10 patients (90%) with relapsed or refractory AML (Table 2). In the responding patients, IC_50_ values ranged between 20 and 450 nM (median: 100 nM) without major differences when comparing freshly diagnosed AML patients with relapsed or refractory AML (Table 2, Figure 2A). In normal BM samples (n=3), JQ1 also produced growth inhibition, but the IC_50_ values were higher compared to that found in AML cells (IC_50_: 100-500 nM, median: 250 nM).

**Table 2:** Effects of JQ1 on proliferation of primary leukemic cells

				^3^H-thymidine-uptake	
AML No#	FAB	WHO	Source PB/BM	+cytokines** IC_50_ (nM)	-cytokines IC_50_ (nM)
1*	M1	AML with t(9;11)	PB	280	n.t.
2	M5	AML monoblastic	BM	90	70
3	M5	AML with (9;11)/NPM1m	BM	160	240
4	M4	AML with mutated NPM1	PB	1,030	420
5	M1	AML with mutated NPM1	BM	420	n.t.
6	M4	AML myelomonocytic	BM	1,420	140
7	M1	AML with myelodysplasia	BM	40	n.t.
8*	M5	AML with myelodysplasia	PB	40	40
9	M5	AML monoblastic	BM	50	50
10	M4	AML with inv16	BM	150	70
11	M2	AML with t(8;21)	BM	40	n.t.
12	M2	AML with t(8;21)	BM	60	n.t.
13	RAEB-T	AML with myelodysplasia	BM	n.t.	60
14	M1	AML without maturation	BM	n.t.	150
15*	sec.	AML with myelodysplasia	PB	n.t.	1,800
16*	M6a	acute erythroid leukemia	PB	40	n.t.
17*	M4	AML myelomonocytic	BM	n.t.	280
19	M4	Therapy related AML	BM	n.t.	250
20	M1	AML with mutated NPM1	BM	n.t.	440
22*	sec.	AML with myelodysplasia	BM	n.t.	70
23*	sec.	AML with myelodysplasia	PB	n.t.	85
24*	M4	AML myelomonocytic	BM	n.t.	230
25*	sec.	AML with myelodysplasia	BM	n.t.	90
26	M4	AML with myelodysplasia	BM	n.t.	260
27*	M4	AML monocytic	BM	n.t.	20
28	M1	AML with mutated NPM1	BM	n.t.	80
29	sec.	AML with myelodysplasia	BM	n.t.	30
30	M5	AML with mutated NPM1	BM	n.t.	110

* These patients were analyzed at relapse or persistence of blasts.

** Cells were cultured in stem cell factor (SCF), interleukin-3 (IL-3) and granulocyte colony-stimulating factor (G-CSF) (each 100 ng/ml).

Abbreviations: AML, acute myeloid leukemia; FAB, French-American-British cooperative study group; PB, peripheral blood; BM, bone marrow; n.t., not tested.

### JQ1 induces apoptosis in primary AML cells

**Figure 3:**
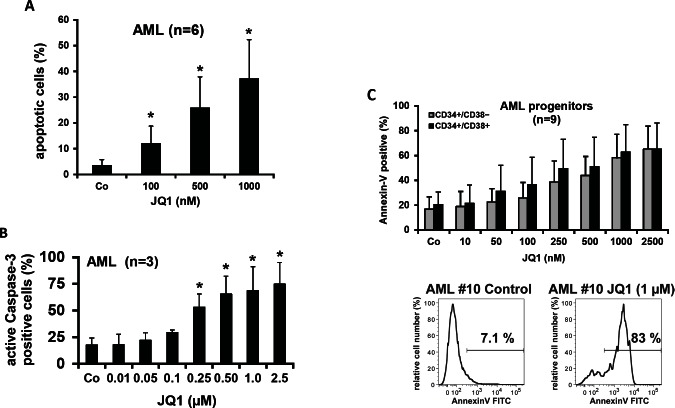
Effects of JQ1 on survival of AML stem- and progenitor cells Primary AML cells were incubated in the absence (Co) or presence of JQ-1 (10–2,500 nM) for 48 hours. Then, the percentage of apoptotic cells was determined on Wright-Giemsa-stained cytospin preparations (A), and the percentage of active Caspase-3-positive cells was determined by flow cytometry (B). Results represent the mean±S.D. from 6 patients (A) or 3 patients (B). Asterisk: p<0.05 compared to medium control. C: Upper panel: Primary AML cells (n=9) were cultured in control medium (Co) or JQ1 (10-2,500 nM) at 37°C for 48 hours. CD34^+^/CD38^+^ AML progenitor cells and CD34^+^/CD38^−^ AML stem cells were examined by combined staining for surface antigens and Annexin-V-FITC. Apoptosis was expressed as percent Annexin-V-positive cells. Results in the upper panel represent the mean±S.D. from 9 donors. The lower panel shows an example of Annexin-V expression in CD34^+^/CD38^−^ AML stem cells after exposure to control medium or JQ1 (1 μM) for 48 hours in patient #10.

In a next step, we examined the mechanism(s) of JQ1-induced growth inhibition in AML cells. In these experiments, JQ1 was found to induce apoptosis in primary AML cells in all samples tested, including cells derived from freshly diagnosed or relapsed patients. Induction of apoptosis was measured by light microscopy (Figure 3A) as well as by caspase 3 staining and flow cytometry (Figure 3B). JQ1 also produced apoptosis in all AML cell lines tested confirming our previous data (not shown) [33].

### JQ1 induces apoptosis in CD34^+^/CD38^+^ and CD34^+^/CD38^−^ leukemic stem- and progenitor cells in freshly diagnosed AML and relapsed/refractory AML

In a next step, we examined the effects of JQ1 on survival of leukemic stem- and progenitor cells derived from our AML patients. As shown in Figure 3C, JQ1 induced apoptosis in CD34^+^/CD38^+^ as well as CD34^+^/CD38^−^ stem and progenitor cells in all samples and all donors tested, including patients with relapsed or refractory AML, and without major differences when comparing FAB or WHO subtypes.

### JQ1 synergizes with ARA-C in producing growth inhibition in AML cells

**Figure 4:**
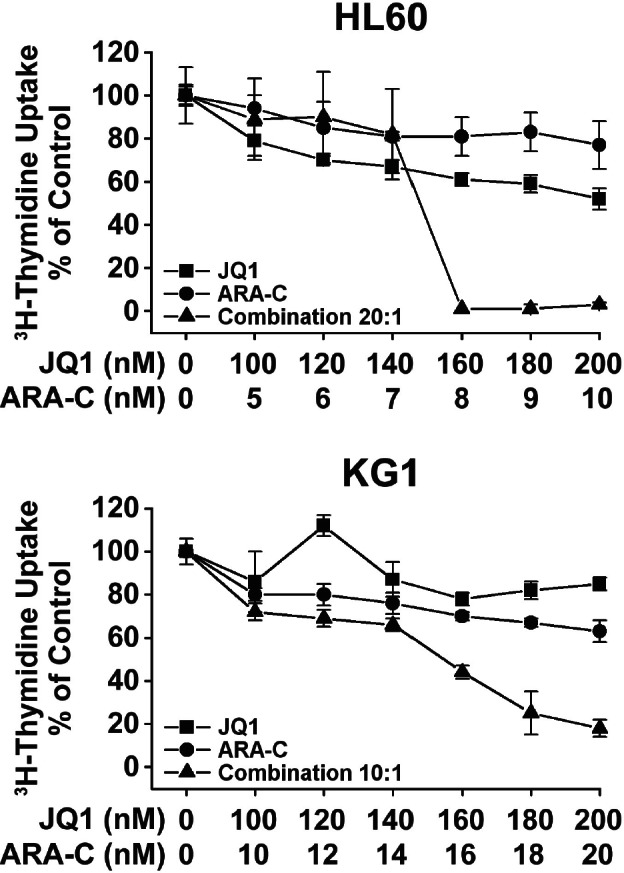
Synergistic growth-inhibitory effects of JQ1 and ARA-C on growth of AML cells HL60 cells (upper panel) and KG1 cells (lower panel) were incubated in control medium or in medium containing various concentrations of JQ1, various concentrations of cytosine arabinoside (ARA-C), or a combination of both drugs at various concentrations at a fixed drug-ratio (20:1 for HL60 cells and 10:1 for KG1). Drugs were applied at 37°C for 48 hours. After incubation, uptake of ^3^H-thymidine was measured. Results are expressed as percent of medium control. Result of one typical experiment are shown. Almost the same results were obtained in 2 other independent experiments.

Since JQ1 and other (similar) BET bromodomain inhibitors are considered to be used in clinical trials in AML patients in combination with conventional cytostatic drugs like ARA-C, we asked whether JQ1 and ARA-C would produce cooperative or even synergistic growth-inhibitory effects on AML cells. For these experiments, the AML cell lines HL60 and KG1 were employed. As visible in Figure 4, JQ1 was found to exert strong synergistic antileukemic effects when combined with ARA-C in both cell lines (Figure 4). These data suggest that drug combinations employing conventional cytostatic drugs, like ARA-C and a BET bromodomain inhibitor, may produce synergistic antileukemic effects in AML cells.

## DISCUSSION

Based on their self-renewal and disease-propagating capacity, leukemic stem- and progenitor cells represent an attractive therapeutic target in AML [26-32]. Notably, AML LSC are considered a relevant source of relapsing disease and exhibit intrinsic as well as acquired resistance [28-32]. Therefore, considerable efforts have been made to understand the mechanisms of LSC resistance, and to identify new drug targets that play an important role in survival, self renewal, and proliferation of AML LSC. However, so far, little is known about the expression of molecular targets in AML LSC and about effects of various targeted drugs [25-28]. We have recently shown that the epigenetic reader BRD4 is a novel promising target in AML [33]. In the present study, we have extended these analyses and show that AML LSC express BRD4, and that the BRD4-targeting drug JQ1 induces growth inhibition and apoptosis in these cells, which may have clinical implications. In addition, our data are the first to show that a drug combination consisting of a BET bromodomain inhibitor and ARA-C, can produce strong synergistic growth-inhibitory effects in AML cells.

Expression of BRD4 in AML stem- and progenitor cells was demonstrable by qPCR and by immunostaining. Both the CD34+/CD38- and the CD34+/CD38+ stem- and progenitor cells contained BRD4 mRNA as well as the BRD4 protein in all categories of AML, including refractory or relapsing disease. In addition, all AML cell lines examined were found to express BRD4. An interesting observation was that normal CD34+ stem cells also expressed BRD4, with similar expression levels compared to LSC. In addition, myeloid progenitor cells and even mature granulocytic cells were found to express BRD4 in the normal BM. These data suggest that targeting of BRD4 may result in a decreased production of normal BM cells. Indeed, we were able to show that JQ1 inhibits the growth of normal BM cells *in vitro*. However, the concentrations required to fully block growth of normal BM cells were higher as that required to block proliferation of AML blasts. This difference may well provide a “pharmacological window” sufficient to introduce JQ1 or similar BET bromodomain inhibitors in clinical trials. In line with this assumption, JQ1 was not found to act as a myelosuppressive agent when applied *in vivo* in various mouse models [33].

BRD4 is a well known transcriptional regulator and chromatin modifier that plays a key role in cell cycle progression in normal and neoplastic cells [34-36]. More recent data suggest, that BRD4 exhibits atypical kinase activity and interacts with a number of nuclear and cytoplasmic interaction-partners such as SPA-1 [37,38]. In the present study, we were able to show that BRD4 is expressed in the cytoplasm and in nuclei of AML blasts and CD34+CD38- AML LSC. The prominent cytoplasmic location of BRD4 was somehow unexpected and suggests that BRD4 may fulfil as yet unknown biological functions in the cytoplasmic compartment of AML cells. An interesting observation was that BRD4 is also expressed in the cytoplasm and nuclei of normal bone marrow cells and highly enriched cord blood progenitor cells, although the expression levels were lower compared to AML cells. However, even in terminally differentiated non-dividing BM cells, BRD4 was found to be expressed in the cytoplasmic and nuclear compartment. These observations suggest that BRD4 may fulfil important functions in normal and neoplastic hematopoiesis, including immature myeloid stem- and progenitor cells and both the nuclear and cytoplamic compartment of these cells.

We have recently shown that the BRD4-blocking drug JQ1 induces growth inhibition and apoptosis in human and murine AML cells [33]. In the present study, we have extended these investigations and asked whether JQ1 would also block the growth and survival of leukemic cells in patients with relapsed or resistant AML. Indeed, our results show that JQ1 induces growth inhibition and apoptosis with reasonable IC50 values in primary AML cells in almost all patients examined, including patients with relapsed or resistant disease. This observation is of particular interest and may have clinical implications. In fact, these patients have a very poor prognosis, and novel drugs will be required to improve therapy. Whether indeed BRD4-targeting agents will be successful in the treatment of AML remains at present unknown. Based on our promising preclinical results, we recommend that BET bromodomain inhibitors should be evaluated for their efficacy in AML in forthcoming clinical trials.

As mentioned above, LSC are a major operational target of drug therapy [26-32,39]. In order to learn whether JQ1 would act anti-leukemic at the LSC level, we analyzed responses of CD34+CD38- cells and CD34+/CD38+ cells to JQ1. In these experiments, JQ1 was found to induce apoptosis of CD34+CD38- and CD34+/CD38+ stem- and progenitor cells in all patients examined, including patients with relapsing or resistant AML. These data suggest that JQ1 may be capable of eradicating LSC, an assumption that needs to be confirmed in further preclinical and clinical studies.

Many of the novel anti-leukemic drugs used to treat refractory AML are applied in combination with other anti-AML agents [40-42]. Therefore, it was of considerable interest to learn whether JQ1 would cooperate or even synergize with established chemotherapeutics when applied on AML cells. In the present study, we were able to show that JQ1 and ARA-C produce clear synergistic effects when used in combination to treat AML cells. This observation is of clinical importance and would be in favor of novel treatment concepts combining JQ1 or similar BRD4 inhibitors and ARA-C in patients with relapsing or refractory AML in clinical trials.

In conclusion, we have identified BRD4 as a novel drug target expressed in AML cells including AML LSC, and that JQ1 is an effective agent capable of blocking growth of AML cells and LSC through BRD4 inhibition. These observations may have clinical implications, as BRD4 blockers like JQ1 and similar agents may soon be applied in clinical trials to treat AML. Whether these drugs will improve therapy in relapsed or resistant AML remains to be determined.

## PATIENTS AND METHODS

### Patients and isolation of AML cells

A total number of 37 patients with AML (females, n=16; males, n=21) were examined. The median age was 59 years (range: 21-85 years). Diagnoses were established according to the proposal of the French-American-British (FAB) cooperative study group [43,44] and the classification of the World Health organization (WHO) [45]. Of the 37 patients, 23 had freshly diagnosed AML, and 14 suffered from refractory or relapsed AML. The patients´ characteristics are shown in Table 3. For control purpose we examined bone marrow (BM) cells obtained from one patient with Non-Hodgkin´s lymphoma without BM involvement, one with idiopathic cytopenia, and one with chronic myeloid leukemia (CML) in major molecular remission. Informed consent was obtained prior to BM puncture in each case. The study was approved by the Institutional Review Board (Ethics Committee) of the Medical University of Vienna. Mononuclear cells (MNC) were prepared using Ficoll and stored in liquid nitrogen until used or were used as freshly prepared MNC. After thawing, the viability of AML cells ranged from 75% to 99% as assessed by trypan blue exclusion.

**Table 3:** Patients´ Characteristics

			Diagnosis			%Blast Cells			
No#	F/M	Age Yrs	FAB	WHO	WBC x109/L	PB	BM	Karyotype	Molecular Defects (Mutations)
1*	F	32	M1	AML with t(9;11)	0.4	80%	73%	46,XX, t(9;11)	MLL1-AF9
2	M	61	M5	AML monoblastic	35.1	33%	72%	46,XY, t(11;17)	MLL1-MSF
3	M	65	M5	AML with t(9;11)/NPM1m	94.8	6%	90%	47,XY, t(9;11),+8	MLL1-AF9, FLT3m, NPM1m
4^Δ^	M	80	M4	AML with mutated NPM1	198.5	25%	52%	46,XY	FLT3m, KIT D816V, NPM1m
5	M	54	M1	AML with NPM1m	361.5	95%	92%	46,XY	FLT3m, NPM1m
6	F	49	M4	AML myelomonocytic	15.4	16%	63%	46,XX	FLT3m, NPM1m, CEBPAm
7	M	67	M1	AML with myelodysplasia	74.6	95%	84%	complex	−
8*^Δ^	F	61	M5	AML with myelodysplasia	82.1	77%	74%	46,XX,del12p,del20q	−
9	F	39	M5	AML monoblastic	37.7	65%	94%	47,XX,t(3;11),+8	−
10^Δ^	F	49	M4	AML with inv16	94.2	47%	57%	46,XX,inv16	FLT3m
11^Δ^	M	58	M2	AML with t(8;21)	100.0	60%	58%	46,XY,t(8;21)	−
12	M	23	M2	AML with t(8;21)	13.7	52%	59%	45,X,-Y, t(8;21)	−
13	M	68	RAEB-T	AML with myelodysplasia	3.5	0%	20%	46,XY	−
14^Δ^	M	21	M1	AML without maturation	133.2	95%	88%	46,XY,add(17p)	−
15*^Δ^	M	49	sec.	AML with myelodysplasia	89.7	85%	n.t.	46,XY	−
16*^Δ^	M	68	M6a	Acute erythroid leukemia	6.1	93%	93%	n.t.	−
17*	M	44	M4	AML myelomonocytic	90.2	91%	92%	complex	−
18	F	41	M4	AML myelomonocytic	2.8	51%	72%	46,XX	−
19	F	72	M4	Therapy-related AML	93.2	62%	74%	46,XX	FLT3m, NPM1m
20	F	52	M1	AML with mutated NPM1	64.1	88%	80%	46,XX	NPM1m
21*^Δ^	F	73	M1	AML without maturation	75.6	73%	84%	46,XX,t(3;?)	FLT3m
22*	F	65	sec.	AML with myelodysplasia	4.2	0%	19%	46,XX	FLT3m
23*	M	70	sec.	AML with myelodysplasia	1.9	25%	15%	complex	−
24*	M	26	M4	AML myelomonocytic	93.9	76%	76%	46,XY	FLT3m
25*	M	71	sec.	AML with myelodysplasia	13.0	77%	46%	46,XY	NPM1m, KIT D816V
26	F	45	M4	AML with myelodysplasia	66.8	53%	66%	46,XX	FLT3m
27*	M	72	M4	AML monocytic	20.3	37%	34%	46,XY,+11,-16	FLT3m
28	M	40	M1	AML with mutated NPM1	202.3	61%	65%	46,XY	NPM1m, FLT3m
29	M	75	sec.	AML with myelodysplasia	18.8	29%	21%	47,XY,+mar	JAK2 V617F, FLT3m
30	F	50	M5	AML with mutated NPM1	50.9	4%	23%	46,XX	NPM1m
31^Δ^	F	65	M4	AML with myelodysplasia	62.2	79%	70%	complex	−
32*^Δ^	F	54	M1	AML without maturation	23.3	81%	n.t.	n.t.	−
33*^Δ^	M	59	sec.	AML with myelodysplasia	34.3	26%	n.t.	47,XY,add(3),+8	−
34^Δ^	F	84	M4	AML myelomonocytic	132.0	8%	29%	46,XX	−
35^Δ^	F	85	M1	AML with myelodysplasia	1.7	0%	33%	46,XX,del5q	−
36^Δ^	M	64	M1	AML with maturation	53.5	94%	84%	46,XY	−
37*^Δ^	M	53	M2	AML with myelodysplasia	2.8	16%	37%	45,XY,-7	−

* These patients were analyzed at relapse or persistence of blasts. Abbreviations: WBC, white blood count; F, female; M, male; Yrs, years; FAB, French-American-British cooperative study group; WHO, World Health Organization; PB, peripheral blood; BM, bone marrow; n.t., not tested; FLT3m, mutated FLT3; NPM1m, mutated NPM1; CEBPAm, mutated CEBPA. ^Δ^These patients were purified for CD34^+^/CD38^−^ and CD34^+^/CD38^+^ subfractions by cell sortin

### Cell lines

AML cell lines used in this study were HL60, U937, KG1, MV4-11, and MOLM-13 (all from the German Collection of Microorganisms and Cell Cultures, Braunschweig, Germany). Cell lines were maintained in RPMI 1640 medium supplemented with 10% fetal calf serum (FCS; PAA laboratories, Pasching, Austria) at 5% CO_2_ and 37°C.

### Immunocytochemistry (ICC) and immunohistochemistry (IHC)

To confirm expression of BRD4 in leukemic cells, ICC and IHC were performed according to published protocols [46-48]. ICC was performed on AML cell lines (U937, HL-60, KG-1, MV4-11, MOLM-13), primary AML MNC, and sorted CD34+/CD38- AML LSC. Cells were spun on cytospin slides and incubated with a polyclonal rabbit anti-human BRD4 antibody (Sigma-Aldrich, St.Louis, MO; work dilution 1:100) for 20 hours. Cells were then washed and incubated with biotinylated goat-anti-rabbit IgG for 30 minutes. As chromogen, alkaline phosphatase complex (Biocare, Walnut Creek, CA) was used. Antibody-reactivity was made visible by Neofuchsin (Nichirei, Tokyo, Japan). In control experiments, the anti-BRD4 antibody was preincubated with control buffer or a BRD4-specific blocking peptide (Bethyl Laboratories, Montgomery, TX) for 1 hour before ICC or IHC were carried out.

IHC was performed on sections prepared from paraffin-embedded, formalin-fixed BM biopsy specimens (AML, n=13; normal/reactive BM: n=6) using the indirect immunoperoxidase staining technique as reported [46-48]. Prior to staining, sections were pretreated by microwave oven. Endogenous peroxidase was blocked by methanol/H_2_O_2_. Slides were incubated with anti-BRD4 antibody (Sigma-Aldrich, work dilution: 1:50; pH 7.5) at 4°C for 20 hours. Then, slides were washed and incubated with biotinylated anti-mouse IgG (Vector, Burlingame, CA) for 30 minutes, washed, and then exposed to Vectastain ABC KIT for 30 minutes. 3-amino-9-ethylcarbazole (AEC, Sigma) was used as chromogen. All slides were counterstained in Mayer´s Hemalaun.

### Evaluation of proliferation by measuring ^3^H-thymidine uptake

Primary cells (MNC) were cultured in 96-well microtiter plates (TPP, Trasadingen, Switzerland) (5-10 × 10^4^ cells/well) in RPMI 1640 medium (PAA laboratories) plus 10% FCS in the absence or presence of JQ1 (10-5,000 nM) at 37°C (5% CO_2_). After 48 hours, 0.5 μCi ^3^H-thymidine was added (16 hours). Cells were then harvested on filter membranes in a Filtermate 196 harvester (Packard Bioscience, Meriden, CT). Filters were air-dried, and the bound radioactivity was measured in a ß-counter (Top-Count NXT, Packard Bioscience). All experiments were performed in triplicates. Proliferation was calculated as percent of medium control, and the inhibitory effects of JQ1 were expressed as IC_50_ values. In a separate set of experiments, AML cell lines (KG-1, HL60) were incubated in various concentrations of JQ1, various concentrations of ARA-C, or a combination of both drugs at fixed ratio of drug-concentrations.

### Flow cytometric evaluation of apoptosis in AML cells

Primary AML cells (n=9) were incubated in RPMI 1640 medium and 10% FCS in the absence or presence of various concentrations of JQ1 (10-5,000 nM) at 37°C (5% CO_2_) for 48 hours. Thereafter, cells were subjected to flow cytometry experiments following published protocols [48-51]. Apoptosis was measured by combined Annexin-V/propidium-iodide staining and caspase-staining. Before being stained with rabbit anti-human monoclonal antibody (mAb) C92-605 (Becton Dickinson Biosciences) directed against active caspase 3, cells were fixed in paraformaldehyde (2%) and permeabilized in methanol at −20°C (15 minutes). Then, cells were washed and analyzed on a FACSCalibur (Becton Dickinson Biosciences). Apoptosis was expressed as percentage of active caspase 3-positive cells.

To study apoptosis in stem- and progenitor cells, CD45^+^/CD34^+^/CD38^+^ BM progenitor cells and CD45^+^/CD34^+^/CD38^−^ BM stem cells were examined for apoptosis by combined staining for surface antigens, 4´,6-diamidino-2-phenylindole (DAPI; Invitrogen, Carlsbad, CA) and Annexin-V-FITC (eBioscience, San Diego, CA) as described [51]. Apoptosis was expressed as percent of Annexin-V+ cells after gating for DAPI-negative cells (early apoptotic cells). The mAb used to identify stem- and progenitor cells were phycoerythrin-labeled CD34 mAb 581, the allophycocyanin-labeled CD38 mAb HIT2, and the peridinin chlorophyll protein-labeled CD45 mAb 2D1 (all from BD Biosciences, San José, CA).

### Purification of CD34^+^/CD38^+^ cells and CD34^+^/CD38^−^ cells by cell-sorting

In 15 patients with AML (patients #4, #8, #10, #11, #14, #15, #16, #21, #31, #32, #33, #34, #35, #36, #37 in Table 3), CD34^+^/CD38^−^ stem cells and CD34^+^/CD38^+^ progenitor cells were highly enriched by mAb and cell sorting essentially as described [50,51]. The purity of the sorted cells amounted to >98%.

### Reverse transcriptase PCR (RT-PCR) and quantitative PCR (qPCR)

PCR experiments were performed on RNA from AML cell lines, unfractionated AML cells, sorted CD34^+^/CD38^+^ and CD34^+^/CD38^−^ AML cells (n=9) and sorted cord blood CD34^+^/CD38^+^ and CD34^+^/CD34^−^ cells pooled from 3 donors. RNA was isolated using RNeasy Mini-Kit (Qiagen, Hilden, Germany). mRNA levels were quantified on a 7900HT Fast Real-Time PCR System from Applied Biosystems (Foster City, CA), using iTaq SYBR Green Supermix with ROX from Bio-Rad (Hercules, CA). *ABL* was employed as a reference gene. Primers used in this study were: hu BRD4-forward: 5'-GCCCGCAAGCTCCA GGATGT-3'; hu BRD4-reverse: 5'-CCTCAGGCTCGTCCGGCATC-3'; hu ABL-forward: 5'-TGTATGATTTTGTGGCCAGTGGAG-3'; and hu ABL-reverse: 5'-GC CTAAGACCCGGAGCTTTTCA-3'.

### Statistical analysis

Differences in growth and apoptosis in drug-exposed cells were determined by appropriate statistical analysis, including the paired student´s t test. Results were considered to be significantly different when the p value was <0.05.
